# Caspase 6/NR4A1/SOX9 signaling axis regulates hepatic inflammation and pyroptosis in ischemia-stressed fatty liver

**DOI:** 10.1038/s41420-023-01396-z

**Published:** 2023-03-28

**Authors:** Mingwei Sheng, Yiqi Weng, Yingli Cao, Chen Zhang, Yuanbang Lin, Wenli Yu

**Affiliations:** 1 Department of Anesthesiology, Tianjin First Central Hospital, Tianjin, China; 2 Department of General Surgery, Tianjin Medical University General Hospital, Tianjin, China

**Keywords:** Inflammatory diseases, Innate immune cells, Acute inflammation

## Abstract

The mechanism of nonalcoholic fatty liver susceptibility to ischemia/reperfusion (IR) injury has not been fully clarified. Caspase 6 is a critical regulator in innate immunity and host defense. We aimed to characterize the specific role of Caspase 6 in IR-induced inflammatory responses in fatty livers. Human fatty liver samples were harvested from patients undergoing ischemia-related hepatectomy to evaluate Caspase 6 expression. in mice model, we generated Caspase 6-knockout (Caspase 6^KO^) mice to investigate cellular and molecular mechanisms of macrophage Caspase 6 in IR-stimulated fatty livers. In human liver biopsies, Caspase 6 expression was upregulated combined with enhanced serum ALT level and severe histopathological injury in ischemic fatty livers. Moreover, Caspase 6 was mainly accumulated in macrophages but not hepatocytes. Unlike in controls, the Caspase 6-deficiency attenuated liver damage and inflammation activation. Activation of macrophage NR4A1 or SOX9 in Caspase 6-deficient livers aggravated liver inflammation. Mechanistically, macrophage NR4A1 co-localized with SOX9 in the nuclear under inflammatory conditions. Specifically, SOX9 acts as a coactivator of NR4A1 to directly target S100A9 transcription. Furthermore, macrophage S100A9 ablation dampened NEK7/NLRP3-driven inflammatory response and pyroptosis in macrophages. In conclusion, our findings identify a novel role of Caspase 6 in regulating NR4A1/SOX9 interaction in response to IR-stimulated fatty liver inflammation, and provide potential therapeutic targets for the prevention of fatty liver IR injury.

## Introduction

Improving the utilization rate of marginal donor liver (e.g., from donors with fatty liver) is an important strategy to overcome the clinical problem of donor shortage [1, 2]. Compared with normal liver, steatotic donor liver is more sensitive to ischemia /reperfusion (IR) injury, and the risk of early postoperative graft dysfunction or even nonfunction is doubled [3]. However, despite the obvious clinical importance, there are no effective intervention strategies to prevent this condition in humans. Thus, it is urgently needed to explore the underlying molecular mechanism and corresponding therapeutics for IR injury in steatotic donor livers.

Caspase 6, a member of the caspase family, has been reported as a non-essential apoptotic executor protein and involved in many biological processes, such as cell apoptosis and proliferation [4]. Recent findings suggest that Caspase 6 can act as a key regulatory molecule to regulate immune responses and cell death [5]. In response to influenza A virus infection, Caspase 6 participated in regulation of inflammasome formation in a non-protease-dependent manner [6]. Zhao et al. [7] found that an AMPK activator inhibited hepatocyte apoptosis by blocking Caspase 6 activity, thereby preventing the progression from fatty liver to nonalcoholic steatohepatitis (NASH) and subsequent hepatocyte death. Moreover, administration of the chemical inhibitor of Caspase 6 greatly restricted adaptive MERS-CoV replication in mice and increased their survival rate from 33.3 to 80% [8]. However, the specific role of macrophage-derived Caspase 6 in IR injury of fatty liver remains unclear.

Nuclear receptor subfamily 4 group A (NR4A) family members include Nur77/NR4A1, Nurr1/NR4A2, and Nor1/NR4A3. As a master factor of stress response, NR4A1 acts as a transcriptional activator or repressor by binding to DNA or proteins and plays critical roles in various pathophysiological processes such as immune regulation, energy metabolism, and tumorigenesis [9, 10]. Genome-wide analysis identified NR4A1 as a key inducer and general mediator of T cell dysfunction, making it a potential target for development of tumor immunotherapy [11]. NR4A1 is lowly or not expressed in macrophages under physiological conditions. However, in response to harmful stimuli, NR4A1 can be activated in various types of macrophages [12]. Previous studies found that the mutation of small ubiquitin-like modifier (SUMO) modification sites in NR4A1 enhanced its stability and the release of inflammatory cytokines from macrophages [13]. Yet, this is no report exploring the relation between NR4A1 and Caspase 6 to affect the inflammatory response of fatty liver IR.

Sex-determining Y box (SOX) genes are widely conserved in mammals and have been involved in biological processes including embryonic development, cell differentiation, and inflammatory responses [14]. Thus far, more than 20 SOX genes have been discovered in vertebrates, with SOX9 being the most extensively studied [15]. Previous study demonstrated that SOX9 attenuated acute kidney injury by regulating Wnt/β-catenin signaling [16]. In contrast, a Tomo-sequencing assay of ischemic myocardium identified collagen I-positive fibroblast-derived SOX9 as the key regulator of IR-induced myocardial fibrosis [17]. Accordingly, the functions of SOX9 in various cell types and cellular activities are still controversial.

Herein, we hypothesized that Caspase 6 is a key component in activating the innate immune response in IR-induced fatty liver. First, liver structure, function, and Caspase 6 expression in patients undergoing partial hepatectomy of fatty livers were evaluated. Second, Caspase 6-knockout (Caspase 6^KO^) mice were employed to investigate the specific role of Caspase 6-mediated innate immune signaling in IR-induced inflammatory responses in fatty livers. Finally, the exact mechanisms by which Caspase 6 regulated inflammasome activation and pyroptosis in macrophages were explored using in vivo and in vitro models.

## Results

### Caspase 6 expression is positively associated with hepatic IR injury in fatty livers

**Fig. 1:**
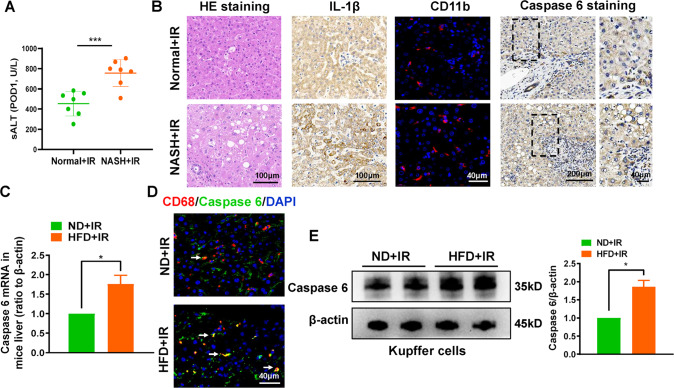
Caspase 6 expression is positively associated with hepatic IR injury in fatty livers. Liver samples were harvested from patients during hepatectomy (after hepatic portal vein occlusion). **A** sALT was detected at the first day postoperatively after hepatectomy, *N* = 7/group; (**B**) H&E staining, IL-1β IHC staining, CD11b IF staining, Caspase 6 IHC staining in human liver biopsies, scale bar: 200 µm, 100 µm, 50 µm; (**C**) qRT-PCR analysis of Caspase 6 in IR-stressed livers with HFD or ND diets; (**D**) IF analysis of AlexaFluor488-labeled Caspase 6 and Cy5-labeled CD68 positive macrophages in ischemic livers, Scale bars, 40 µm; (**E**) WB-assisted Caspase 6 expression profile in Kupffer cells isolated from mice ischemic livers. *N* = 4–7/group, all data represent the mean ± SD, *p < 0.05, **p < 0.01, ***p < 0.001.

To verify the severity of the effect of IR on fatty liver, liver structural and functional damage indexes were detected in both liver biopsies and serum samples from patients who underwent partial hepatectomy with or without NASH. Compared with healthy livers, IR-stressed fatty livers showed a significant increase in serum ALT in patients with NASH (Fig. 1A), accompanied by greater histopathological damage, proinflammatory mediator release, and macrophage activation (Fig. 1B). Next, the upregulated Caspase 6 expression was observed in patients‘ fatty liver samples compared with samples without fatty livers (Fig. 1B). Similarly, Caspase 6 mRNA expression was also enhanced in the fatty livers subjected to IR insult (Fig. 1C). Double-immunofluorescence staining showed that increased Caspase 6 was mainly concentrated in liver macrophages (Kupffer cells) (Fig. 1D), which was further confirmed by detecting Caspase 6 protein levels in isolated liver macrophages (Kupffer cells) using western blotting assay (Fig. 1E). These data suggest that Caspase 6 plays a critical role in IR injury of fatty livers.

### Caspase 6 deletion alleviates liver damage and inflammatory response during fatty liver IR injury

**Fig. 2:**
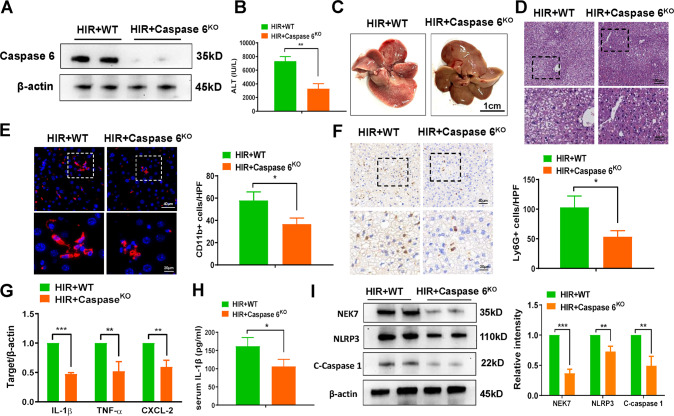
Caspase 6 deletion alleviates liver damage and inflammatory response during fatty liver IR injury. WT and Caspase 6^KO^ mice were used to establish fatty liver IR (HIR) models. Samples were harvested after 90 min ischemia and 6 h of reperfusion. **A** Liver Caspase 6 expression was evaluated by Western blot assay. **B** sALT level was detected in ischemic livers; (**C**) Representative images of livers after IR surgery, scale bar: 1 cm; (**D**) Representative H&E staining in ischemic liver tissue. scale bars: 100 μm, 40 μm; (**E**) IF staining and quantification of CD11b + macrophages in ischemia livers, scale bars: 40 μm, 20 μm; (**F**) IHC staining and quantification of Ly6G+ neutrophiles in ischemia livers, scale bars: 40 μm, 20 μm; (**G**) Detection of cytokines IL-1β, TNF-α and CXCL-2 by qRT-PCR in ischemic livers; (**H**) ELISA analysis of serum IL-1β levels; (**G**) Western blotting analysis and relative intensity of NEK7, NLRP3 and C-Caspase 1. *N* = 4–7/group, all data represent the mean ± SD, *p < 0.05, **p < 0.01, ***p < 0.001.

On the basis of the expression of Caspase 6 being upregulated in IR-stressed fatty livers, we hypothesized a functional association of Caspase 6 during fatty liver IR injury. Conventional Caspase 6^KO^ mice were generated using the CRISPR/Cas9 system. As expected, the expression of Caspase 6 was not detectable in Caspase 6^KO^ mice compared with WT mice (Fig. 2A). After 60 min of ischemia and 6 h of reperfusion, Caspase 6 deletion significantly reduced the elevated levels of serum ALT after fatty liver IR insult compared with the levels in WT mice (Fig. 2B). Assessment of liver morphology after IR surgery revealed that Caspase 6^KO^ fatty livers exhibited reduced edema, sinusoidal congestion, vacuolization and local necrosis (Fig. 2C, D). What’s more, CD11b + macrophage accumulation (Fig. 2E) and neutrophil activation (Fig. 2F) were also relatively suppressed in IR-induced Caspase 6^KO^ fatty liver. The mRNA expression of proinflammatory factors IL-1β, TNF-α, and CXCL-2 (Fig. 2G), as well as serum IL-1β concentrations (Fig. 2H) were decreased in ischemic fatty livers from Caspase 6^KO^ mice. Western blot analysis also showed that NEK7, NLRP3 and C-Caspase 1 protein expressions were downregulated in IR-induced Caspase 6^KO^ fatty liver (Fig. 2I). Given that Caspase 6 deficiency ameliorated fatty liver damage subjected to IR surgery, we tested whether the liver protection of Caspase 6 deletion come from alleviating IR stress or liver steatosis in advance. Caspase 6 siRNA was injected in NASH mice 24 h before establishing liver IR model. As expected, serum ALT levels, histopathological damage, and release of liver proinflammatory factors were simultaneously inhibited after Caspase 6 siRNA injection 24 h before establishing the fatty liver IR model (Supplementary Fig. 1). Together, these results demonstrated that Caspase 6 deletion ameliorated liver damage and inflammatory induced by fatty liver IR treatment.

### Caspase 6 activates NR4A1/SOX9 signaling and induces S100A9 expression in IR-stressed fatty liver

**Fig. 3:**
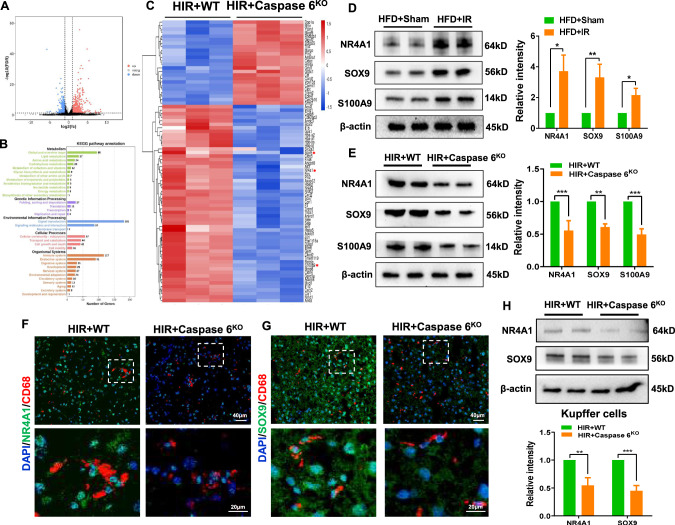
Caspase 6 activates NR4A1/SOX9 signaling and induces S100A9 expression in IR-stressed fatty liver. WT and Caspase 6^KO^ mice subjected to fatty liver IR (HIR) were collected to perform RNA-sequencing analysis. **A** Volcano Plot displaying 615 genes to be upregulated and 440 genes downregulated. **B** KEGG pathway enrichment analysis of major biological pathways contributing to Caspase 6 function. **C** Heatmap showing the expression of Top 80 different genes. **D**, **E** Western-assisted analysis of NR4A1, SOX9 and S100A9; (**F**, **G**) IF analysis of AlexaFluor488-labeled NR4A1 or SOX9 and Cy5-labeled CD68 positive macrophages in ischemic livers, Scale bars, 40 µm, 20 µm; (**H**) WB-assisted NR4A1 and SOX9 expression profile in Kupffer cells isolated from mice ischemic fatty livers of WT and Caspase 6^KO^ mice. *N* = 4–7/group, all data represent the mean ± SD, *p < 0.05, **p < 0.01, ***p < 0.001.

To investigate the underlying mechanism of the Caspase 6-dependent immune regulation during fatty liver IR, RNA-Sequencing with IR-challenged fatty livers from WT and Caspase 6^KO^ mice was performed. Interestingly, we detected changes in 1054 genes, of which 440 were downregulated and 614 were upregulated (Fig. 3A). Kyoto Encyclopedia of Genes and Genomes (KEGG) pathway analysis revealed “Immune system”, “Signal transduction” and “Cellular community” as the top pathways regulated by Caspase 6 (Fig. 3B). Among the top 80 differentially expressed genes in terms of fold change (Fig. 3C), changes of SOX9, NR4A1, and S100A9 aroused our attention. We found that IR triggered the upregulation of SOX9, NR4A1 and S100A9 in fatty liver (Fig. 3D), which could be suppressed by Caspase 6 deficiency (Fig. 3E) in line with the RNA-sequencing results. IF staining suggested that Caspase 6 deficiency reduced the localization of both NR4A1 and SOX9 in CD11b-positive macrophages (Fig. 3F), which was further confirmed by western blot assay of NR4A1 and SOX9 in Kupffer cells isolated in ischemic fatty livers from WT and Caspase 6^KO^ mice (Fig. 3G).

### NR4A1/SOX9 signaling controls proinflammatory responses in IR-stressed fatty liver

**Fig. 4:**
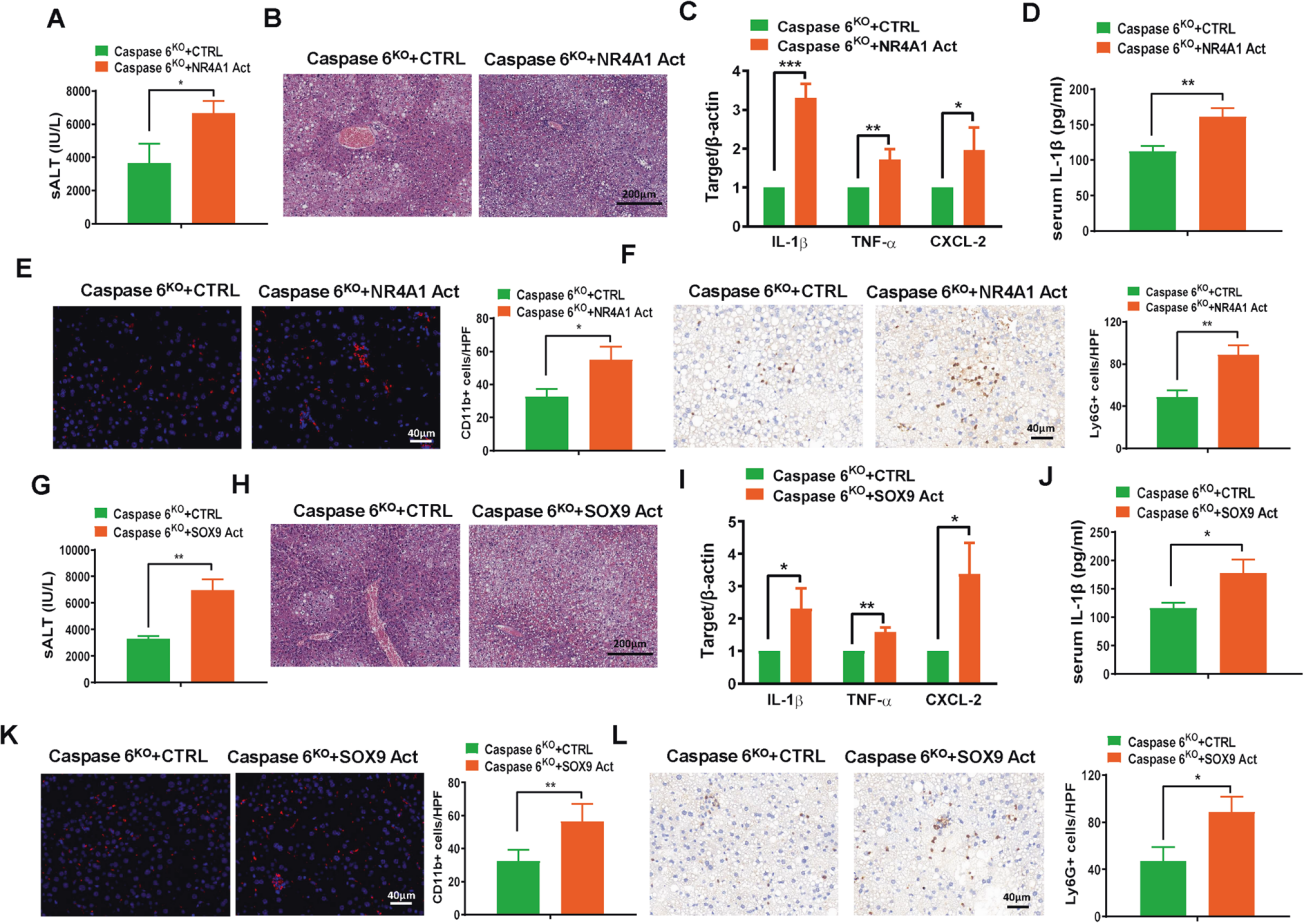
NR4A1/SOX9 signaling controls proinflammatory responses in IR-stressed fatty liver. NR4A1 or SOX9 activation (Act) plasmids or control (CTRL) vectors mixed with mannose-conjugated polymers were injected into Caspase 6^KO^ mice 24 h before establishing HIR models. **A**, **G** Serum ALT levels in ischemic fatty livers; (**B**, **H**) H&E staining of ischemic fatty livers, Scale bar: 200 μm; (**C**, **I**) Detection of cytokines IL-1β, TNF-α and CXCL-2 by qRT-PCR in ischemic fatty livers; (**D**, **J**) ELISA analysis of serum IL-1β levels; (**E**, **K**) IF staining and quantification of CD11b^+^ macrophages in ischemic fatty livers, Scale bar: 40 μm; (**F**, **L**) IHC staining and quantification of Ly6G^+^ neutrophils in ischemic fatty livers, Scale bar: 40 μm; *N* = 4–7/group, all data represent the mean ± SD, *p < 0.05, **p < 0.01, ***p < 0.001.

Given that Caspase 6 deficiency inhibited the NR4A1/SOX9 signaling, we next explored whether NR4A1/SOX9 signaling affected the downstream proinflammatory signaling cascade in IR-induced fatty liver. As previously reported, mannose-coupled vectors can be delivered to macrophages/Kupffer cells by expressing a mannose-specific membrane receptor. The transfection efficiency of NR4A1/SOX9 activation plasmids was verified by IF staining and western blotting analysis (Supplementary Fig. 2). Compared with Caspase 6^KO^ + CTRL group, NR4A1-activation aggravated liver histopathology and functional damage, as evidenced by elevated ALT and greater necrotic area in ischemic fatty liver from Caspase 6^KO^ mice (Fig. 4A, B). Moreover, mRNA expression of proinflammatory factors coding for IL-1β, TNF-α, and CXCL-2 (Fig. 4C), serum concentrations of IL-1β (Fig. 4D), the accumulation of CD11b-postive macrophages (Fig. 4E), and Ly-6G-positive neutrophils (Fig. 4F) were decreased in Caspase 6^KO^ + NR4A1-Activation group compared with those in Caspase 6^KO^ + CTRL group. Similarly, macrophages overexpressing SOX9 antagonized the liver protective effect of Caspase 6 deletion by promoting the inflammatory response and pathological damage (Fig. 4G–L).

### NR4A1 interacts with SOX9 in nuclear to target S100A9 transcription in macrophages

**Fig. 5:**
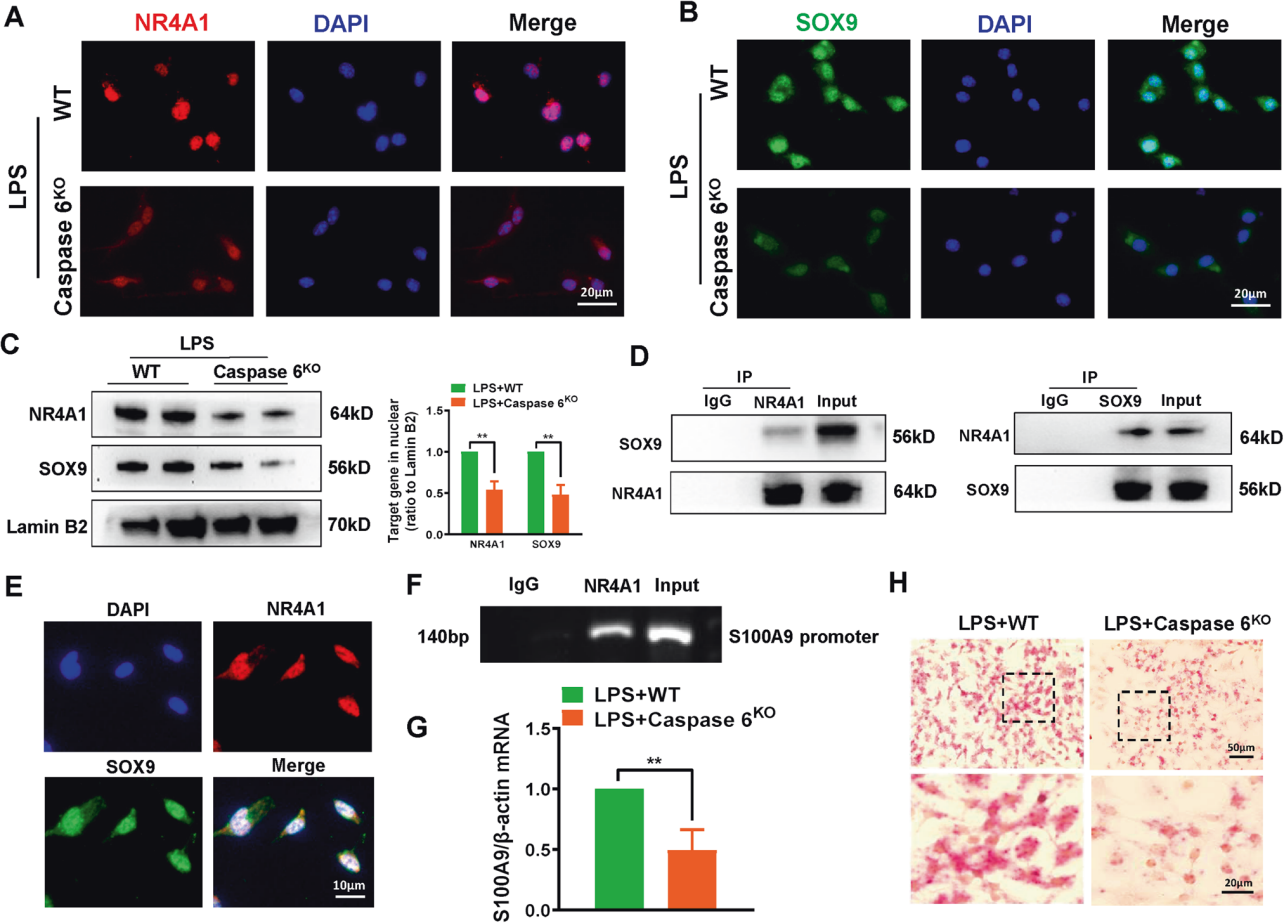
NR4A1 interacts with SOX9 in nuclear to target S100A9 transcription in macrophages. Bone marrow-derived macrophages (BMMs) derived from WT or Caspase 6^KO^ mice were treated with LPS for 6 h. IF staining of NR4A1 (**A**) and SOX9 (**B**) in LPS-stimulated macrophages. DAPI was used to visualize nuclei (blue). Scale bars: 20 μm. **C** Western-assisted analysis of NR4A1 and SOX9 in nuclear extracts; (**D**) Immunoprecipitation analysis of NR4A1 and SOX9 in LPS-stimulated macrophages from WT and Caspase 6^KO^ mice. **E** IF staining for macrophage NR4A1 (red) and SOX9 (green) co-localization in the nuclear after LPS stimulation, scale bar: 10 μm; (**F**) ChIP assay with macrophages using anti-NR4A1 antibody in LPS-treated BMMs; (**G**) RNA-ISH detecting S100A9 mRNA in macrophages by using specific probe; (**H**) Detection of S100A9 mRNA by qRT-PCR in macrophages. *N* = 4–7/group, all data represent the mean ± SD, *p < 0.05, **p < 0.01, ***p < 0.001.

Having demonstrated that Caspase 6 activated NR4A1/SOX9 signaling in IR-induced fatty liver inflammation, we analyzed the interaction between NR4A1 and SOX9 in macrophages. As expected, IF staining revealed that Caspase 6 deletion markedly inhibited nuclear localization of both NR4A1 (Fig. 5A) and SOX9 (Fig. 5B) in LPS-induced macrophages compared with WT group. Western blotting assay further confirmed that the nuclear expressions of NR4A1 and SOX9 were reduced in Caspase 6^KO^ macrophages (Fig. 5C). Strikingly, co-immunoprecipitation results revealed that SOX9 bound endogenous NR4A1 in LPS-challenged BMMs (Fig. 5D). IF staining confirmed that NR4A1 could colocalize with SOX9 in the nuclear (Fig. 5E). To decipher the potential mechanism by which Caspase 6 regulates NR4A1/SOX9-mediated inflammatory responses, we performed ChIP with LPS-stimulated BMMs. The data showed that NR4A1 localized to the S100A9 promoter region (Fig. 5F), suggesting that the transcription of S100A9 is directly regulated by NR4A1. In line with the ChIP results, both RNA-ISH (Fig. 5G) and qRT-PCR assay (Fig. 5H) proved that Caspase 6 deficiency decreased the mRNA expression of S100A9 in LPS-stimulated macrophages. In conclusion, these results confirm that NR4A1 targeting the transcription of S100A9 through interaction with SOX9 is critical for Caspase 6-controlled immune regulation in macrophages.

### SOX9 is necessary for NR4A1 to mediate S100A9 transcription in macrophages during Caspase 6-mediated inflammation

**Fig. 6:**
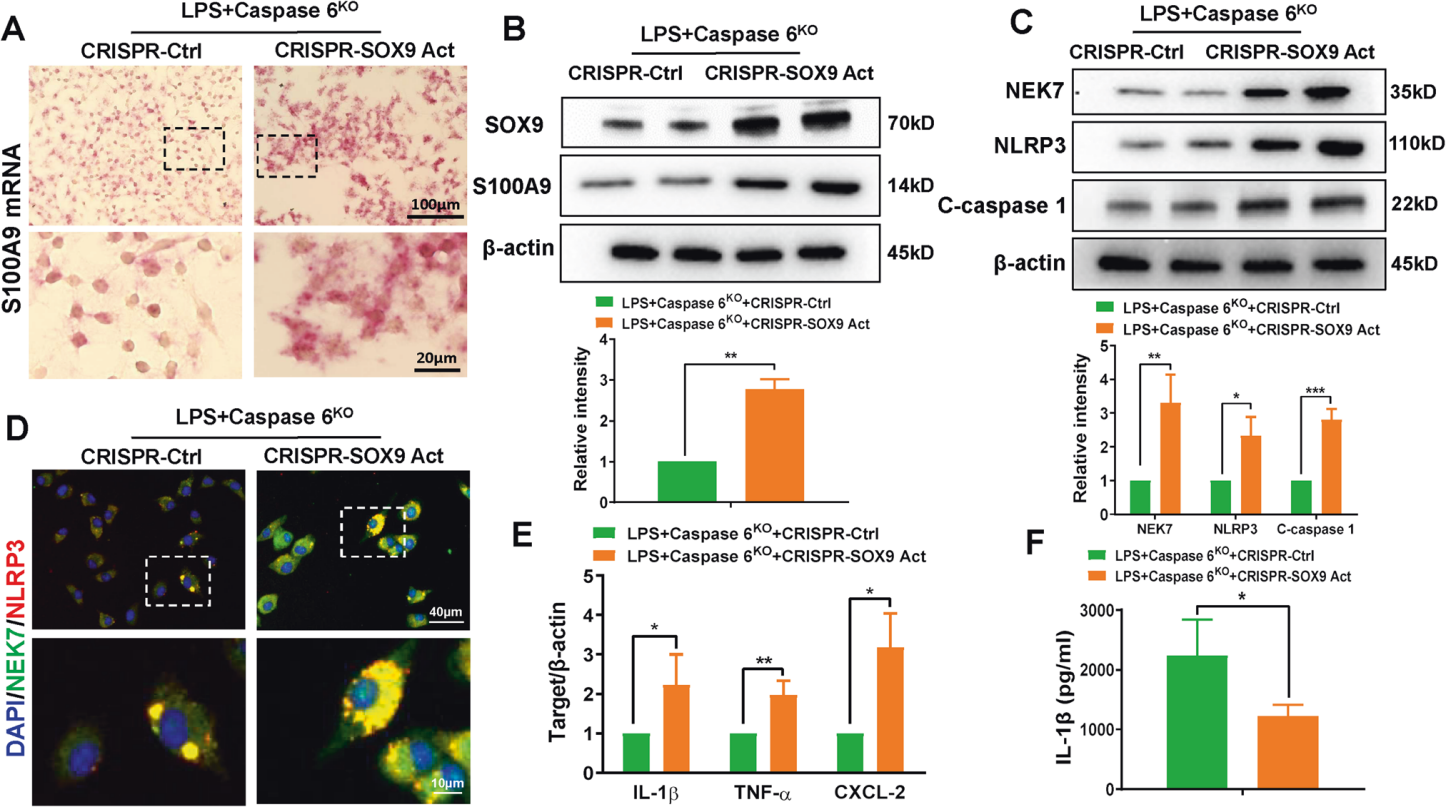
SOX9 is necessary for NR4A1 to mediate S100A9 transcription in macrophages during Caspase 6-mediated inflammation. BMMs were isolated from Caspase 6^KO^ and WT mice and transfected with the p-CRISPR-SOX9 activation or control vector followed by LPS stimulation. **A** RNA-ISH detecting S100A9 mRNA in macrophages by using specific probe; (**B**) Western blotting and relative intensity of SOX9 and S100A9; (**C**) Western blotting and relative intensity of NEK7, NLRP3 and C-caspase 1; (**D**) IF staining for NEK7 and NLRP3 expression in macrophages. DAPI was used to visualize nuclei. Scale bars: 40 μm, 10 μm; (**E**) Detection of cytokines IL-1β, TNF-α and CXCL-2 by qRT-PCR in macrophages; (**F**) ELISA analysis of IL-1β levels in culture medium. *N* = 4–7/group, all data represent the mean ± SD, *p < 0.05, **p < 0.01, ***p < 0.001.

To clarify the role of SOX9 in regulating S100A9 transcription, BMMs were isolated from Caspase 6^KO^ mice and transfected with CRISPR-mediated SOX9 activation plasmids. RNA-ISH assays confirmed that Caspase 6 deficiency inhibited S100A9 mRNA, whereas CRISPR-mediated SOX9 activation reduced S100A9 transcription in Caspase 6^KO^ macrophages (Fig. 6A). Western blot results further confirmed that SOX9 activation increased S100A9 protein expression (Fig. 6B). Moreover, compared with CRISPR-Ctrl group, the CRISPR-mediated SOX9 activation plasmids increased expression of NEK7, NLRP3, and C-caspase 1 (Fig. 6C). IF staining was further confirmed the enhanced co-localization of NEK7 and NLRP3 in SOX9-activated macrophages induced by LPS (Fig. 6D). mRNA levels of proinflammatory factors IL-1β, TNF-α, and CXCL-2 (Fig. 6E), and secretion of IL-1β were also relatively increased (Fig. 6F), suggesting that SOX9 is a key mediator for NRA1 to induce S100A9 activation and inflammation in macrophages.

### S100A9 is essential for NLRP3 activation and pyroptosis in Caspase 6-mediated inflammation induced by IR in fatty liver

**Fig. 7:**
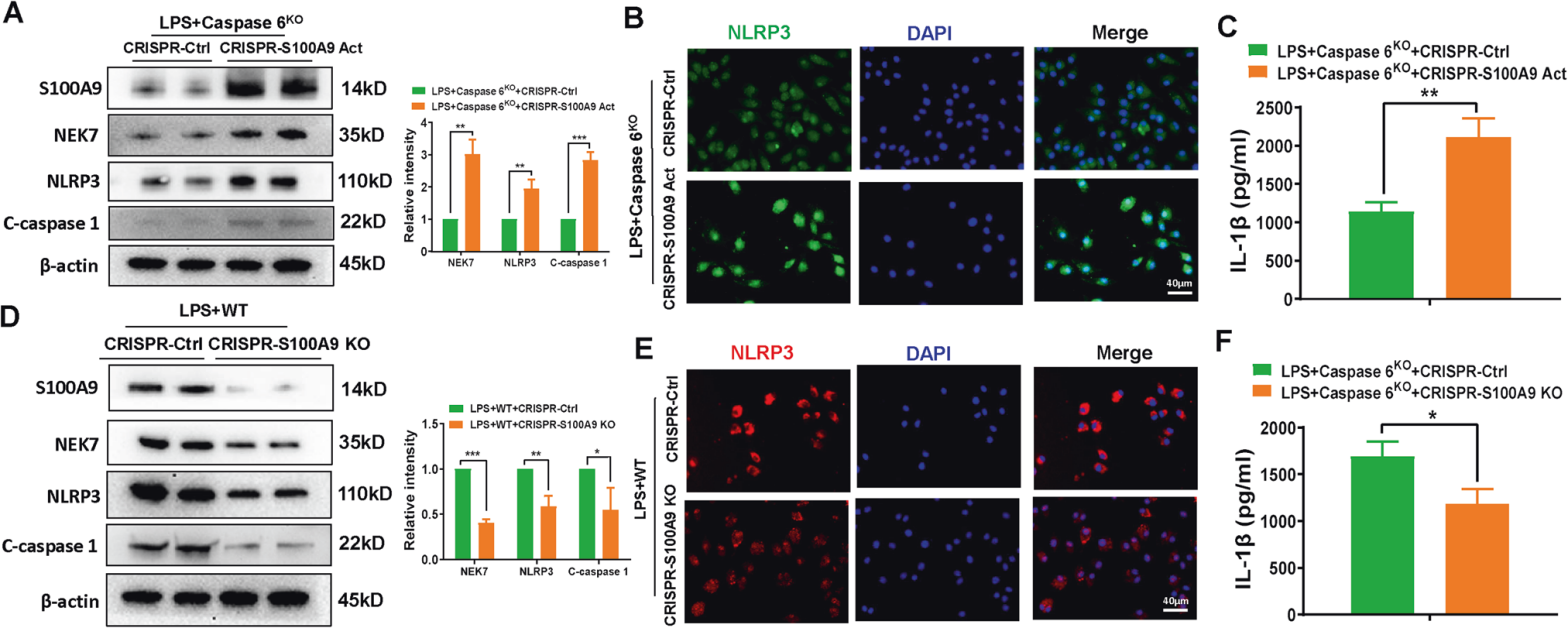
S100A9 is essential for NLRP3 activation and pyroptosis in Caspase 6-mediated inflammation induced by IR in fatty liver. BMMs from WT or Caspase 6^KO^ mice were transfected with the CRISPR-S100A9 Act, CRISPR-S100A9 KO or control vector followed by LPS stimulation; (**A**, **D**) Western blotting and relative intensity of S100A9, NEK7, NLRP3 and C-caspase 1 in LPS-stimulated BMMs; (**B**, **E**) IF staining for NLRP3 expression in macrophages. DAPI was used to visualize nuclei. Scale bar: 40 μm; (**C**, **F**) ELISA analysis of IL-1β levels in culture medium. *N* = 4–7/group, all data represent the mean ± SD, *p < 0.05, **p < 0.01, ***p < 0.001.

Having determined that NR4A1/SOX9 axis targeted S100A9 transcription in macrophages during Caspase 6-mediated immune regulation, we next examined whether S100A9 could affect NLRP3 inflammasome activation and macrophage pyroptosis. BMMs isolated from WT and Caspase 6^KO^ mice were subsequently transfected with CRISPR-mediated S100A9 activation vector or S100A9-KO vector. CRISPR-mediated activation of S100A9 promoted expression of NEK7, NLRP3, and C-caspase 1 in LPS-stimulated macrophages extracted from Caspase 6^KO^ mice (Fig. 7A). IF staining further revealed that S100A9 activation aggravated NLRP3 expression (Fig. 7B) and IL-1β release in macrophages (Fig. 7C). In contrast, the CRISPR-S100A9-KO vector inhibited NEK7/NLRP3 function, C-caspase 1 expression (Fig. 7D, E), and IL-1β release from macrophages (Fig. 7F). Collectively, these in vitro results strongly suggest that Caspase 6-mediated NEK7/NLRP3 inflammasome activation and pyroptosis are dependent on S100A9 function.

## Discussion

To the best of our knowledge, this study reveals that macrophage Caspase 6 can activate NR4A1/SOX9 signaling to regulate S100A9 transcription in response to IR-stimulated fatty liver inflammation. Importantly, we found that Caspase 6 promoted upregulation of SOX9 and NR4A1 expression in the nuclear, which subsequently induced the interaction between NR4A1 and SOX9 to mediate the transcription of the downstream target gene S100A9, ultimately leading to NEK7/NLRP3 inflammasome activation and pyroptosis in macrophages in fatty liver following IR. Our results highlight the importance of macrophage NR4A1-SOX9-S100A9 axis as a critical regulator in Caspase 6 regulation of innate immune responses in IR-induced fatty liver injury.

Fatty liver, a common type of marginal donor liver, is more sensitive to IR injury than healthy liver. It is an emerging risk factor of delayed or even loss of graft function after liver transplantation [18]. However, intracellular and molecular mechanisms of IR injury in fatty liver are poorly understood. Macrophages are the first defenders against harmful stimuli in the body [19]. However, macrophage overactivation may trigger innate immune activation by releasing a variety of danger-associated molecular patterns including HMGB1, which leads to strong inflammation response and further hepatocytes apoptosis/necrosis [20]. Single-cell RNA-sequencing analysis has proved that transplanted fatty donor liver exhibited proinflammatory macrophages accumulation compared with non-fatty donor liver [21]. In consistent with previous studies, IR stress aggravated fatty liver damage in both human and mice liver samples, as evidenced by the upregulation of serum ALT levels and increased histopathological injury. What’s more, the activation of macrophages and release of the proinflammatory factors were significantly increased.

The Caspase family can be divided into two categories: regulators of apoptosis and inducers of inflammatory cell death, which plays critical roles in regulation of programmed cell death and inflammatory actions [4]. Although being regarded as an apoptosis-related protein, accumulating evidence has suggested that Caspase 6 is a key regulator of innate immunity, inflammasome activation, and host defenses [5]. In the current study, endogenous Caspase 6 was significantly increased in IR-stressed fatty liver of both human and mice samples. Furthermore, IR-induced Caspase 6 expression was mainly accumulated in macrophages but not hepatocytes, suggesting a key role of macrophage-derived Caspase 6 in inflammatory response undergoing fatty liver IR. To clarify the functional role of Caspase 6, Caspase 6^KO^ mice and in vivo macrophage targeted Caspase 6-siRNA injection were introduced to assess the degree of liver damage to ischemic fatty liver. As expected, Caspase 6 deletion significantly ameliorated structural and functional damage after fatty liver IR surgery, as evidence by downregulation of serum ALT levels, histopathological damage, macrophage/ neutrophil infiltration, and the expression of proinflammatory cytokines. Moreover, NEK7/NLRP3 inflammasome activation and C-caspase 1 expression were significantly inhibited in Caspase 6-deficient fatty liver induced by IR stress. Together, our findings demonstrate that deletion of macrophage-derived Caspase 6 attenuates innate immune activation and NLRP3 inflammasome activation during fatty liver IR.

It has been well documented that Caspase 6-mediated innate immune regulation is associated with a variety of signal transduction pathways [22]. Our RNA-sequencing data showed that Caspase 6 deficiency induced the changes of 1054 genes in ischemic fatty liver, which were involved in Lipid metabolism, Signal transduction, and the Immune system pathway. Among the top 80 differentially expressed genes, the changes of NR4A1 and SOX9 attracted our attention. NR4A1, belonging to NR4A family members, is widely expressed in organs or tissues. Previous study showed that activation of NR4A1 was an endogenous modulator of transforming growth factor β1 signaling [23]. During the progression of fatty liver, NR4A1 could disrupt ATP production by aggravating mitochondrial fission and Reactive oxygen species (ROS) liberation [24]. However, depletion of NR4A1 in B cells markedly promoted cholesterol and triglyceride levels and contributed to atherosclerosis in mice [25], indicating that the role of NR4A1 is “bidirectional”. Consistent with our RNA-sequencing results, western blotting analysis showed that IR activated the NR4A1 expression in fatty liver, which was reversed by Caspase 6 knockdown. What’s more, both IF staining and western blotting assay confirmed that Caspase 6 deficiency could inhibit NR4A1 expression in liver macrophages. Furthermore, in vivo transfection of NR4A1-activation plasmid into macrophages showed that NR4A1 overexpression antagonized the liver protective effects of Caspase 6 deficiency. Thus, our findings demonstrate that Caspase 6 activates macrophage NR4A1 expression to subsequently inhibit inflammatory responses in IR-induced fatty liver.

We also found that IR stress could increase SOX9 expression in ischemic fatty liver. Both RNA-sequencing and western blot analysis confirmed that Caspase 6 deletion mediated the downregulation of SOX9 in fatty liver following by IR. Similar to NR4A1, SOX9 is also a transcription factor that plays vital roles in sexual differentiation, liver fibrosis and tissue repair [26, 27]. Previous studies indicated that deletion of a partial enhancer of SOX9 could lead to complete XY male-to-female sex reversal [28]. In mice model of acute myocardial infarction, specific inhibition of SOX9 in fibroblasts weaken myocardial inflammation and fibrosis by reducing leukocyte infiltration [29]. Similarly, our data reveal that macrophage SOX9 knockdown inhibited hepatic structural and functional damage and also the production of proinflammatory mediators in IR-challenged fatty liver. Collectively, these results suggest that SOX9 is also a crucial signaling molecule for Caspase 6-mediated regulation of IR-induced inflammatory response in fatty liver.

Having clarifying the critical functions of both NR4A1 and SOX9 in macrophages during regulation of inflammatory action induced by fatty liver IR, we next examined what molecular mechanism confers the ability of NR4A1/SOX9 to regulate Caspase 6-controlled innate immune activation? He et al. identified the reciprocal feedback regulation between YAP and NR4A1 in the process of liver regeneration [30]. In both mouse and human liver tumors, YAP-SOX9 signaling determined hepatocyte plasticity and lineage-specific hepatocarcinogenesis [31]. Therefore, it can be deduced that the cross-communication occurs between NR4A1 and SOX9. Our in vitro data demonstrated that Caspase 6 depletion inhibited the translocation of NR4A1 and SOX9 into nuclear in macrophages. Further evidence confirmed the co-localization of NR4A1 and SOX9 in response to LPS stimulation. Notably, NR4A1 interacted with SOX9 through direct binding. Furthermore, ChIP data showed that NR4A1 could promote transcription by binding to the promoter region of S100A9, suggesting it as the downstream target gene of NR4A1 regulated by Caspase 6. Furthermore, disrupting SOX9 signaling decreased S100A9 expression, inactivated the NEK7/NLRP3 inflammasome, and inhibited pyoptosis in macrophages, indicating that SOX9 acts as a transcriptional coactivator of NR4A1 in Caspase 6-mediated immune regulation.

Another surprising finding is that S100A9 is key to controlling NLRP3 function and pyroptosis. S100A9, also known as myeloid-related protein 14 (MRP14), is a calcium binding protein belonging to the S100 family. S100A9 has been implicated in the regulation of a diverse range of cellular processes including inflammatory actions and cell death [32]. Recent studies have proved that S100A9-triggered cardiac inflammatory injury by inducing macrophages M1 polarization [33]. In addition, airway inflammation induced by the S100A9/nuclear factor-κB signaling cascade was the key mechanism responsible for smoking-related chronic obstructive pulmonary disease [34]. In line with previous reports, knockdown of macrophage S100A9 inhibited NEK7/NLRP3 inflammasome activation and C-caspase 1 expression, which was accompanied by decreased IL-1β release. Similarly, S100A9 activation increased NEK7, NLRP3, and C-caspase 1 levels in Caspase 6-deficient macrophages stimulated by LPS. Taken together, these results reveal the key role of macrophage S100A9 in regulating Caspase 6-mediated hepatic inflammatory injury.

**Fig. 8:**
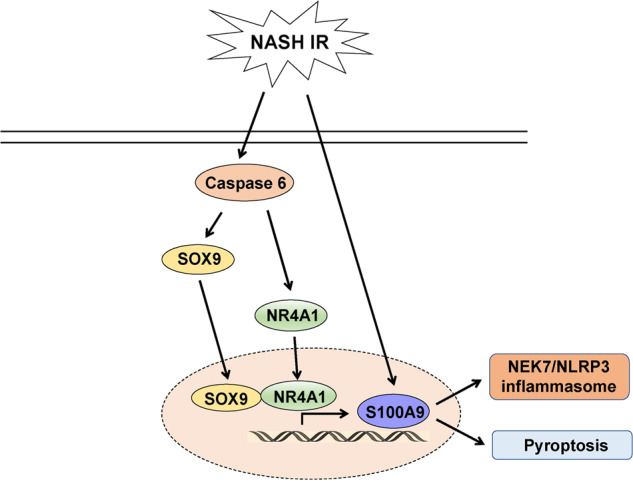
The schematic figure depicts putative molecular mechanisms in Caspase 6-mediated inflammation induced by IR in fatty liver. Caspase 6 controls NR4A1-SOX9 interaction to drive liver inflammatory response. Specifically, SOX9 acts as a coactivator of NR4A1 to target the downstream gene S100A9, a key regulator of NEK7/NLRP3 inflammasome activation and pyroptosis.

Overall, we identify a novel role of Caspase 6 in regulation of NEK7/NLRP3 function and pyroptosis in fatty livers subjected to IR surgery. We confirm that Caspase 6 controls NR4A1-SOX9 interaction to drive liver inflammatory response. Specifically, SOX9 acts as a coactivator of NR4A1 to target the downstream gene S100A9, a key regulator of NEK7/NLRP3 inflammasome activation and pyroptosis (Fig. 8). Our findings provide potential therapeutic targets for the prevention of fatty liver IR injury.

## Methods

### Patients and clinical samples

This study was approved by the Academic Committee of Tianjin Medical University General Hospital (Tianjin, China). Human samples were obtained from 14 patients with benign liver tumors complicated with or without fatty livers undergoing partial hepatectomy (January 2021 to June 2022, Department of General Surgery, Tianjin Medical University General Hospital, Supplementary Table 1). Liver samples were obtained during reperfusion after liver resection (15*–*30 min of ischemia, followed by 15–20 min of reperfusion before abdominal closure). Liver injury was assessed by measuring serum levels of alanine aminotransferase (ALT) on the first postoperative day. Informed consent was obtained from all participants.

### Fatty liver IR model and treatments

Caspase 6 was knocked out in C57 mice by CRISPR/Cas9 technology (Cyagen Bioscience, Jiangsu, China). All animal experimental protocols were approved by the Animal Ethics Committee of Tianjin First Central Hospital. Liver steatosis was induced in 4-week-old male wild-type (WT) mice or Caspase 6^KO^ mice fed a high-fat diet (HFD) (Supplementary Table 2 for details) or normal diet (ND) for 12 weeks. Subsequently, mouse liver IR model was established according to our previous study [35]. Briefly, the artery/portal vessels were clipped for 90 min and reperfusion was initiated by releasing the clamp. Liver tissue and serum were harvested 6 h after reperfusion. The same procedure was performed in the Sham groups without blocking the blood vessels. Alexa Fluor™ 488-labeled control vectors, Caspase 6-knockdown siRNA, and NR4A1 or SOX9 activation plasmids (2 mg/kg; Santa Cruz Biotechnology) were mixed with mannose-conjugated polymers (Polyplus-transfection, France) at a ratio according to the manufacturer’s instructions. Then they were injected through tail vein of mice 24 h before IR surgeries as described in our previous report [36]. Mice were then randomly divided into groups without blinding in different settings.

### ALT detection

Serum ALT levels were detected using an ALT kit (Sigma Aldrich) according to the manufacturer’s instructions.

### Enzyme-linked immunosorbent assay (ELISA)

Interleukin 1β (IL-1β) levels in serum or culture medium were evaluated according to commercially available ELISA kits (PI301, Beyotime).

### Histopathology

Livers were fixed with 4% formalin for 24 h and subsequently embedded in paraffin and cut into 5-μm-thick sections. For liver histopathology, samples were stained with hematoxylin and eosin (H&E). For immunohistochemical (IHC) staining, samples were dehydrated, exposed to antigen, and then incubated with IL-1β (ab283818, Abcam, 1:100 dilution), Caspase 6 (ab185645, Abcam, 1:100 dilution), and Ly-6G (ab261916, Abcam, 1:100 dilution) antibodies respectively at 4 °C overnight. For immune-fluorescence (IF) staining, tissue sections or cultured cells were fixed with 4% formalin for 30 min and then incubated at 4 °C overnight with antibodies against CD11b (ab184308, Abcam, 1:100 dilution), CD68 (#26042, Cell signaling Technology, 1:100 dilution), Caspase 6 (ab185645, Abcam, 1:100 dilution), NR4A1 (ab153914, Abcam, 1:100 dilution); SOX9 (ab185966, Abcam, 1:100 dilution), NEK7 (sc-393539, Santa Cruz Biotechnology, 1:100 dilution), and NLRP3 (ab270449, Abcam, 1:100 dilution). Then samples were incubated with the secondary antibody conjugated to Alexa Fluor 488 (Jackson Immunoresearch) or Alexa Fluor Cy5 (Jackson Immunoresearch) for 2 h at room temperature in the dark. Immunofluorescence images were captured using a fluorescence microscope (Keyence BZ-X810, Osaka, Japan).

### Quantitative real-time PCR

Total RNA was extracted with Trizol reagent (15596026, Invitrogen) and detected by real-time PCR. RNA was reverse transcribed into cDNA using PrimeScript RT kit (A15300, Invitrogen). qPCR experiments were performed using a SYBR Green PCR kit (4367659, Applied biosystems). β-actin was used as an internal control. Primer sequences of target genes including Caspase 6, IL-1β, TNF-α, CXCL-2, S100A9, and β-actin are listed in Supplementary Table 3.

### Western blot analysis

Tissues or cells were equilibrated in immunoprecipitation assay buffer at 4 °C for 30 min. The supernatant was collected and centrifuged at 12,000 × *g* for 20 min. After separating proteins by polyacrylamide gel electrophoresis, they were transferred to polyvinylidene fluoride (PVDF) membranes and incubated with antibodies against Caspase 6 (ab185645, Abcam, 1:1000 dilution), NEK7 (sc-393539, Santa Cruz Biotechnology, 1:1000 dilution), NLRP3 (ab270449, Abcam, 1:1000 dilution), cleaved Caspase 1 (C-caspase 1) (#89332, Cell signaling Technology, 1:1000 dilution), NR4A1 (ab153914, Abcam, 1:1000 dilution), SOX9 (ab185966, Abcam, 1:1000 dilution), S100A9 (ab242945, Abcam, 1:1000 dilution), and β-actin (#4970, Cell signaling Technology, 1:2000 dilution). β-actin was used as an internal reference. The nuclear and cytosolic fractions were prepared with NE-PER Nuclear and Cytoplasmic Extraction Reagents (ThermoFisher Scientific). Lamin B2 (#12255, Cell signaling Technology, 1:1000 dilution) was used as an internal reference of nuclear protein. IBright FL1000 (Invitrogen, Carlsbad, CA, USA) was used to analyze the expression of target proteins. Full and uncropped western blots have been shown in Supplementary Material (2).

### Isolation of primary Kupffer cells

Primary Kupffer cells were isolated from mice according to our previous methods [35]. Briefly, mouse livers were digested with preheated EGTA and 0.75 g/L collagenase type I solution at 37 °C. Liver non-parenchymal cells were obtained by centrifugation at 50 × *g* for 5 min and resuspended in Hank’s balance solution. Kupffer cells were isolated using a 50%/25% two-step Percoll gradient solution (1500 × *g* at 4 °C for 15 min). Kupffer cells located in the middle of centrifuge tubes were collected and resuspended in Dulbecco’s Modified Eagle Medium (DMEM) and 10% fetal bovine serum (FBS). Non-adherent cells were removed by culture medium exchange.

### Bone marrow-derived macrophages (BMMs) isolation and transfection in vitro

BMMs extracted from the femur and tibia of mice were filtered through a 200-μm nylon cell filter. After centrifugation at 300 × *g* for 10 min, cell (1×10^6^/well) were resuspended in 15% L929-conditioned medium and 10% FBS. After 7d of culture, cells were transfected with CRISPR-SOX9 activation, CRISPR-S100A9 activation, CRISPR/Cas9-S100A9 KO, or control vector (Santa Cruz Biotechnology). After 48 h, transfected cells were exposed to lipopolysaccharide (LPS) for 6 h.

### RNA-sequencing assay

Liver tissues harvested from fatty liver IR model of WT mice or Caspase 6^KO^ mice were subjected to RNA-sequencing analysis (*n* = 3/group). Total RNA was extracted with Trizol reagent separately. The cDNA libraries were constructed using the NEBNext® UltraTM RNA Library Prep Kit for Illumina® (NEB, USA) according to the manufacturer’ instruction. Pathway analysis was used to figure out the significant pathways of the differential genes according to KEGG database.

### Protein immunoprecipitation assay

After lysing cells with NP-40 lysis buffer (50 mM Tris pH7.4, 10 mM EDTA, 150 mM NaCl, 1% NP-40), total cell isolates were incubated with anti-SOX9 (sc-166505, Santa Cruz Biotechnology) or anti-NR4A1 (sc-365113, Santa Cruz Biotechnology) antibodies overnight at 4 °C, followed by protein-G/A Beads for 4 h at 4 °C. The protein was separated by heating at 95 °C for 5 min. Supernatants were collected and analyzed by standard immunoblot analysis.

### Chromatin immunoprecipitation (ChIP) assay

ChIP experiments were performed with a ChIP kit (ab185913, Abcam). Briefly, BMMs were treated with 1% formalin for 15 min to crosslink protein and chromatin. The reaction was then terminated by adding 0.125 M glycine for 5 min. Cells were washed and resuspended in ChIP lysis buffer for 15 min. Cell lysates were centrifuged and resuspended in nuclear lysis buffer. After 15 min of ultrasonic oscillation, chromatin was analyzed on 2% agarose gel for DNA fragment length analysis. Chromatin was subsequently immunoprecipitated overnight with anti-NR4A1 antibody (12235-1-AP, Proteintech), while a normal IgG antibody was used as a negative control. Antibody/chromatin samples were mixed with Protein A Sepharose beads. After repeated cleaning and purification, the obtained DNA products were tested by PCR. Primer sequences for the NR4A1-reactive S100A9 promoter region are listed in Supplementary Table 3.

### RNA in situ hybridization (RNA-ISH)

RNA ISH was detected using an RNAscope System according to the manufacturer’s protocol (Advanced Cell Diagnostics, Newark, CA, USA). Mouse S100A9 probes, as well as negative and positive control probes, were purchased from Advanced Cell Diagnostics. In general, BMMs on glass slides were fixed with 10% neutral buffered formalin and subsequently dehydrated with 50%, 70%, and 100% ethanol for 5 min. Slides were then dried at room temperature for 5 min and treated with hydrogen peroxide for 10 min, followed by protease IV for 30 min. Next, slides were incubated with probes in a HybEZ oven at 40 °C for 2 h. Conditions included treatment at 40 °C for 30 min (AMP1, AMP3, and AMP5) or 20 min (AMP2, AMP4, and AMP6). Finally, the RNA signal was detected by incubation with Fast Red for 10 min. Eosin was used to count cells, and slides were left to dry at 60 °C for 15 min. Finally, RNA-ISH staining images were captured using a light microscope.

### Statistical analysis

SPSS22.0 software was used for statistical analysis. All data are expressed as mean ± standard deviation (SD). Comparisons between groups were performed using ANOVA or *t* test. A two-sided *P* < 0.05 was considered statistically significant. Data are shown as representative of at least four independent experiments.

## Supplementary information

Reply to email confirming the agreement of the addition to author list

Supplementary material (1)

Original Data File

## Data Availability

The mRNA expression profile data are available in the Figshare databases (Digital Object Identifier: 10.6084/m9.figshare.21981866). Other datasets used in the current study are available from the corresponding author on reasonable request.
