# Supplementary material for: Caspase 6/NR4A1/SOX9 signaling axis regulates hepatic inflammation and pyroptosis in ischemia-stressed fatty liver
Source: Cell Death Discov. 2023 Mar 28;9:106. doi: 10.1038/s41420-023-01396-z (PMC10043527; doi:10.1038/s41420-023-01396-z)
Supplement: Supplementary file 1 — Reply to email confirming the agreement of the addition to author list [file 41420_2023_1396_MOESM1_ESM.pdf]

The agreement of the addition to the author list in the manuscript

- Manuscript Number: CDDISCOVERY-23-0094R1
- Title: Caspase 6/NR4A1/SOX9 signaling axis regulates hepatic inflammation and pyroptosis in ischemia-stressed fatty liver
- Authors: Wenli Yu, Mingwei Sheng, Yiqi Weng, Yingli Cao, Chen Zhang, and Yuanbang Lin

The request agreement from co-authors addition to the manuscript.

The agreement of the addition to the author list in the manuscript (CDDISCOVERY-23-0094R1)

yuwenli999 于2023年3月3日 星期五 下午15:44 发送给 cyingli95...

发送状态 全部发送成功 (查看详情)

Journal: Cell Death Discovery
Manuscript Number: CDDISCOVERY-23-0094R1
Title: Caspase 6/NR4A1/SOX9 signaling axis regulates hepatic inflammation and pyroptosis in ischemia-stressed fatty liver
Authors: Wenli Yu, Mingwei Sheng, Yiqi Weng, Yingli Cao, Chen Zhang, and Yuanbang Lin

Dear Yingli Cao,

The corresponding author professor Wenli Yu has listed you as a contributing author of the following submission via Elsevier's online submission system.

Submission Title: Caspase 6/NR4A1/SOX9 signaling axis regulates hepatic inflammation and pyroptosis in ischemia-stressed fatty liver

Elsevier asks all authors to verify their co-authorship by confirming agreement to publish this article if it is accepted for publication.

Please read the following statement and confirming that you agree to the addition into the author list of the submitted manuscript.

I irrevocably authorize and grant my full consent to the corresponding author of the manuscript to: (1) enter into an exclusive publishing agreement with Elsevier on my behalf (or, if the article is to be published under a CC BY license, a non-exclusive publishing agreement), in the relevant form set out at www.elsevier.com/copyright ; and (2) unless I am a US government employee, to transfer my copyright or grant an exclusive license of rights (or for CC BY articles a non-exclusive license of rights) to Elsevier as part of that publishing agreement , effective on acceptance of the article for publication. If the article is a work made for hire, I am authorized to confirm this on behalf of my employer. I agree that the copyright status selected by the corresponding author for the article if it is accepted for publication shall apply and that this agreement is subject to the governing law of the country in which the journal owner is located.

The response from Yingli Cao

Re: The agreement of the addition to the author list in the manuscript (CDDISCOVERY-23-0094R1)

发件人: "cyingli95" <cyingli95@163.com>
时 间: 2023年3月3日 星期五 下午16:17
收件人: "yuwenli999" <yuwenli999@sina.com>
大 小: 12.24K

Yes! I verify the co-authorship and agree to the addition into the author list of manuscript (CDDISCOVERY-23-0094R1).

---- Replied Message ----

From yuwenli999@sina.com
Date 03/03/2023 15:44
To cyingli95<cyingli95@163.com>
Subject The agreement of the addition to the author list in the manuscript (CDDISCOVERY-23-0094R1)

Journal: Cell Death Discovery
Manuscript Number: CDDISCOVERY-23-0094R1
Title: Caspase 6/NR4A1/SOX9 signaling axis regulates hepatic inflammation and pyroptosis in ischemia-stressed fatty liver
Authors: Wenli Yu, Mingwei Sheng, Yiqi Weng, Yingli Cao, Chen Zhang, and Yuanbang Lin

Dear Yingli Cao,

The corresponding author professor Wenli Yu has listed you as a contributing author of the following submission via Elsevier's online submission system.

Submission Title: Caspase 6/NR4A1/SOX9 signaling axis regulates hepatic inflammation and pyroptosis in ischemia-stressed fatty liver

The response from Chen Zhang

搜索邮件、通讯录、网盘文件等

yuwenli999@sina.com(0) 首页 升级邮箱 手机版 设置 帮助 [退出]

通讯录 邮箱网盘 应用 收件夹 F+会员 Re:The

回复 回复全部 转发 删除 举报 移动 标记 更多 下一封

Re:The agreement of the addition to the author list in the manuscript (CDDISCOVERY-23-0094R1)

发件人: "**zhangchen201608**" <zhangchen201608@163.com> 查看 拒收

时　间: 2023年3月3日 星期五 下午16:30

收件人: "**yuwenli999**" <yuwenli999@sina.com>

大　小: 12.82K

精简信息

Yes, I confirm that I agree to the addition into the author list of the submitted manuscript (Caspase 6/NR4A1/SOX9 signaling axis regulates hepatic inflammation and pyroptosis in ischemia-stressed fatty liver).

--

清晨的香樟树

邮箱: zhangchen201608@163.com

At 2023-03-03 15:53:12, yuwenli999@sina.com wrote:

> Journal: Cell Death Discovery
> Manuscript Number: CDDISCOVERY-23-0094R1
> Title: Caspase 6/NR4A1/SOX9 signaling axis regulates hepatic inflammation and pyroptosis in ischemia-stressed fatty liver
> Authors: Wenli Yu, Mingwei Sheng, Yiqi Weng, Yingli Cao, Chen Zhang, and Yuanbang Lin
>
> Dear Chen Zhang,
>
> The corresponding author professor Wenli Yu has listed you as a contributing author of the following submission via Elsevier's online submission system.

1:1
