# Supplementary material for: Caspase 6/NR4A1/SOX9 signaling axis regulates hepatic inflammation and pyroptosis in ischemia-stressed fatty liver
Source: Cell Death Discov. 2023 Mar 28;9:106. doi: 10.1038/s41420-023-01396-z (PMC10043527; doi:10.1038/s41420-023-01396-z)
Supplement: Supplementary file 2 — Supplementary material (1) [file 41420_2023_1396_MOESM2_ESM.pdf]

Supplementary Material (1)

Caspase 6/NR4A1/SOX9 signaling axis regulates hepatic inflammation and pyroptosis in ischemia-stressed fatty liver

Mingwei Sheng[1], Yiqi Weng[1], Yingli Cao[1], Chen Zhang[1], Yuanbang Lin[2*], Wenli Yu[1*]

[1]Department of Anesthesiology, Tianjin First Central Hospital, Tianjin, China.

[2]Department of General Surgery, Tianjin Medical University General Hospital, Tianjin, China.

***Corresponding author**: Wenli Yu, MD, PhD., Department of Anesthesiology, Tianjin First Central Hospital, Fukang Road NO. 24, Tianjin, PR China, 300192. Email: yuwenli999@sina.com. Yuanbang Lin, MD, PhD., Department of General Surgery, Tianjin Medical University General Hospital, Anshan Road NO. 154, Tianjin, PR China, 300052. Email: linyuanbang@tmu.edu.cn.

Supplementary Table 1. Patient characteristics

**Clinical manifestation**	Patients with NASH	Patients without NASH
Age, years (mean ± SD)	49.01±3.36	50.29±6.02
Gender, n (M/F)	2/5	3/4
BMI, kg/m^2	52.22±3.01	38.08±5.76
Hyperlipidemia (%)	5(71)	0 (0)
**Laboratory tests** (mean ± SD)		
ALT, U/L	825.21 ± 33.90	601.16± 17.08
AST, U/L	595.71 ± 32.59	384.63 ± 19.26
TG, U/L	4.22 ± 0.69	1.92 ± 0.88
TC, U/L	8.17 ± 0.45	5.09 ± 0.31
**Histologic characteristics**		
NAS (mean ± SD)	5.92 ± 0.49	0
Steatosis grade 1/2/3	1/3/3	
Lobular inflammation 0/1/2/3	0/2/3/1	
Ballooning 0/1/2	0/5/2	
Fibrosis stage 0/1/2/3/4	0/1/3/2/1	

BMI, Body Mass Index; ALT, Alanine aminotransferase; AST, Aspartate aminotransferase; TG, Triglyceride; TC, Total cholesterol; NAS, NAFLD Activity Score.

Supplementary Table 2. Composition of high-fat diet and standard diet fed to male C57 mice for 8 weeks

Ingredient	High-fat(g/kg)	Standard(g/kg)
Corn Starch	72	575
Yolk powder	50	0
Lard	200	0
Dextrin	220	185
Sucrose	102	100
Casein	180	153
Cholesterol	20	0
Sodium cholate	2	0
Cellulose	43	80
Mineral mix	55	45
Vitamin mix	12	10
L-Cystine	4	2
Energy(kcal/g)	4.71	4.34
Protein(kcal/g)	20	15
Fat(kcal/g)	45	75
Carbohydrate(kcal/g)	35	10

Supplementary Table 3: Primer sequences for the amplification (H, denotes human, M, denotes mice and p denotes promoter)

Target genes	Forward primers	Reverse primers
M-Caspase 6	5’-AAGTGTTCGATCCAGCCGAG-3’	5’- CAGGTTGTCTCTGTCTGCGT-3’
M-IL-1β	5’- TGTAATGAAAGACGGCACACC-3’	5’-TCTTCTTTGGGTATTGCTTGG-3’
M-TNF-α	5’-GCTACCAAACTGGATATAATCAGGA-3’	5’-CCAGGTAGCTATGGTACTCCAGAA-3’
M-CXCL-2	5'-ACTTCAAGAACATCCAGAG-3'	5'-CTTTCCAGGTCAGTTAGC-3'
M-S100A9	5'-ACCACCATCATCGACACCTTC-3'	5'- AAAGGTTGCCAACTGTGCTTC-3'
M-p-S100A9	5'-TGGAAGACCCACCATAGCCA-3'	5'- TGGCCAATGCAAAGGGTACA -3'
M-β-actin	5’-GTGACGTTGACATCCGTAAAGA-3’	5’-GCCGGACTCATCGTACTCC-3’

Supplementary Figure 1

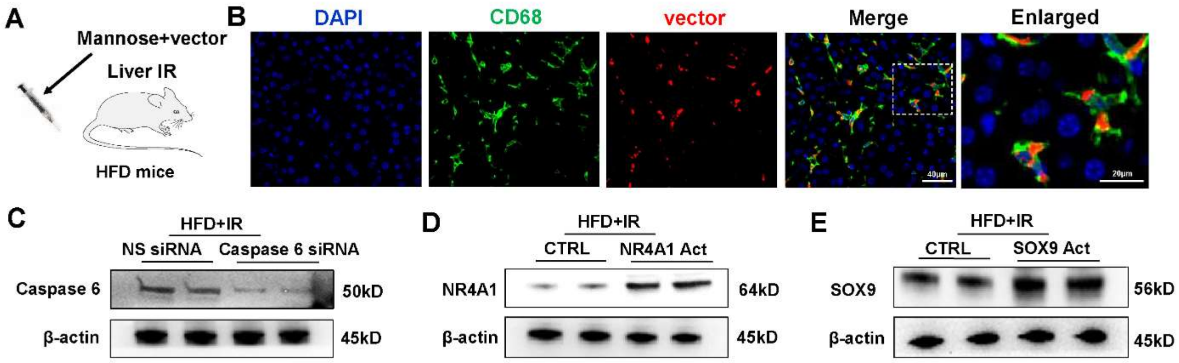

Suppl. Fig. 1. Mannose-conjugated vector was delivered to macrophages/Kupffer cells specifically in IR-stressed fatty liver. (A) Schematic figure for the injection of mannose-conjugated Caspase 6 siRNA, CRISPR-NR4A1 Activation (Act) vector, CRISPR-SOX9 Act vector or Control (CTRL) vector into ischemic fatty livers of Caspase 6^{KO} mice. (B) IF staining of AlexaFluor488-labeled CD68 positive macrophages and AlexaFluor Cy5-labeled vectors in ischemic livers, Scale bars, 40μm, 20μm. The expressions of Caspase 6 (C), NR4A1 (D) and SOX9 (E) was evaluated in Kupffer cells isolated from IR-induced fatty livers by Western blot assay. N=4–6 mice/group, all data represent the mean ± SD, $*p < 0.05$, $**p < 0.01$, $***p < 0.001$.

Supplementary Figure 2

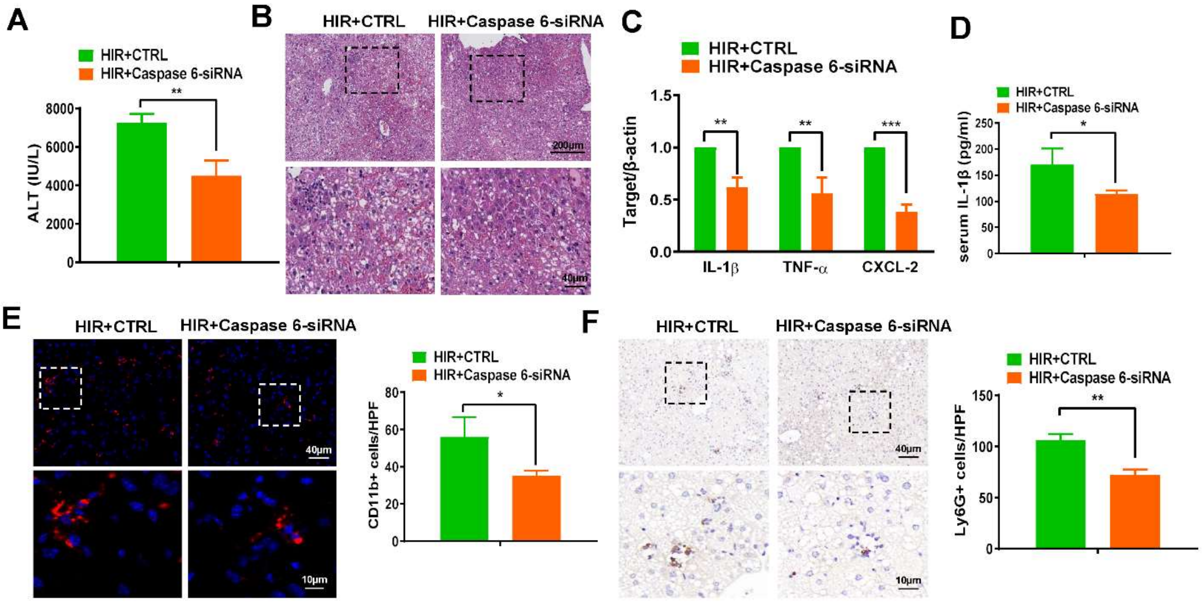

Suppl. Fig. 2. Administration of Caspase 6 siRNA rescues liver damage and inflammation action in IR-stressed fatty liver. Caspase 6-siRNA or control (CTRL) vectors mixed with mannose-conjugated polymers were injected into WT mice fed with high fat diet 24h before establishing liver IR models. (A) Serum ALT levels in ischemic fatty livers; (B) H&E staining of ischemic fatty livers, Scale bar: 200μm, 40μm; (C) Detection of cytokines IL-1β, TNF-α and CXCL-2 by qRT-PCR in ischemic fatty livers; (D) ELISA analysis of serum IL-1β levels; (E) IF staining and quantification of $CD11b^+$ macrophages in ischemic fatty livers, Scale bar: 40μm; (F) IHC staining and quantification of $Ly6G^+$ neutrophils in ischemic fatty livers, Scale bar: 40μm; N=4–6 mice/group, all data represent the mean ± SD, $*p < 0.05$, $**p < 0.01$, $***p < 0.001$.
