# Supplementary material for: Caspase 6/NR4A1/SOX9 signaling axis regulates hepatic inflammation and pyroptosis in ischemia-stressed fatty liver
Source: Cell Death Discov. 2023 Mar 28;9:106. doi: 10.1038/s41420-023-01396-z (PMC10043527; doi:10.1038/s41420-023-01396-z)
Supplement: Supplementary file 3 — Original Data File [file 41420_2023_1396_MOESM3_ESM.pdf]

Fig.1E

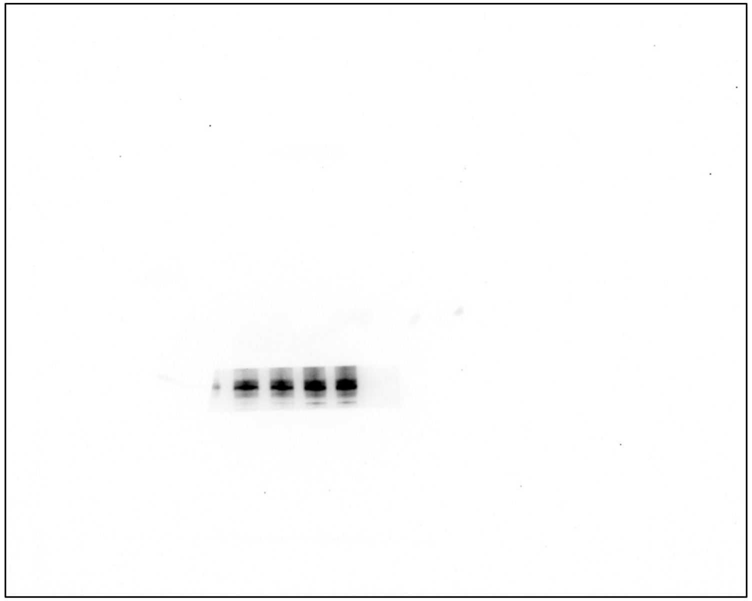

Caspase-6

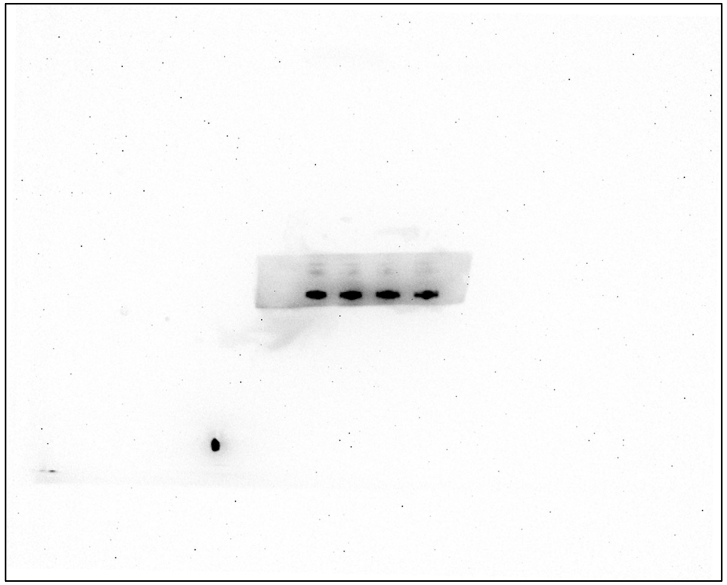

β-actin

Fig.2A

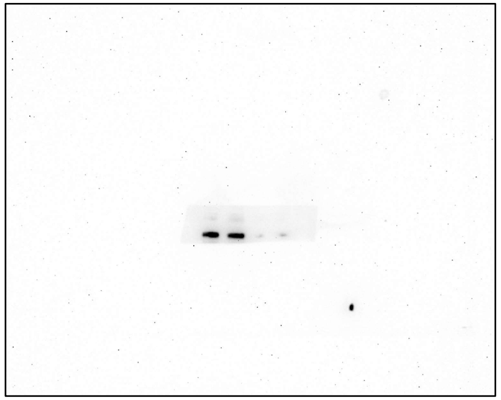

Caspase 6

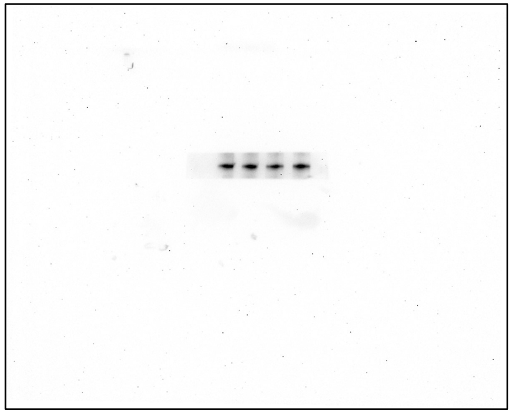

β-actin

Fig.2I

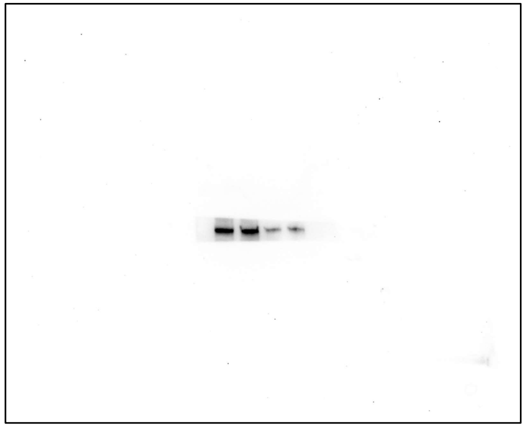

NEK7

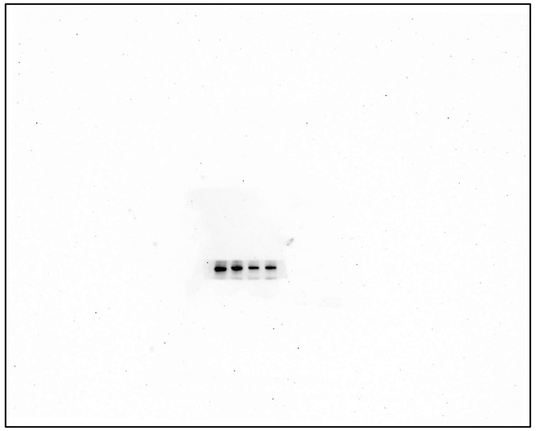

NLRP3

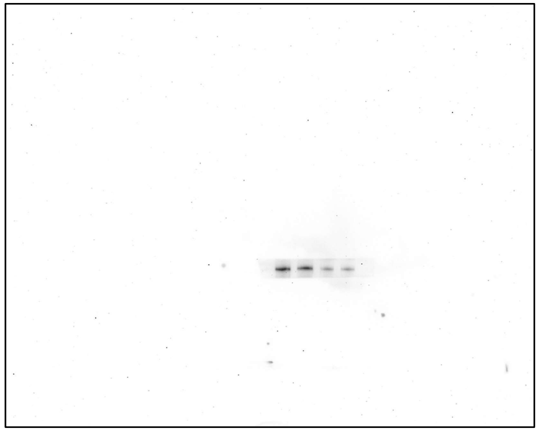

C-Caspase 1

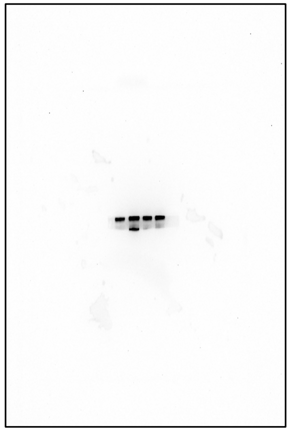

β-actin

Fig.3D

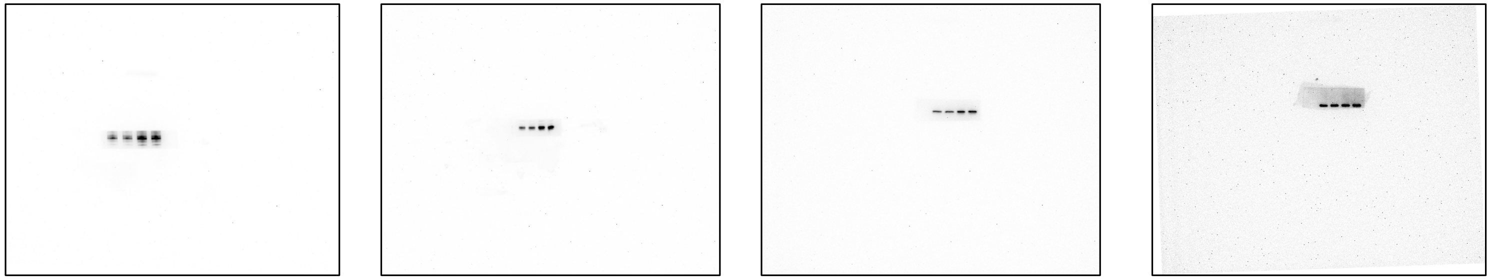

NR4A1 SOX9 S100A9 β-actin

Fig.3E

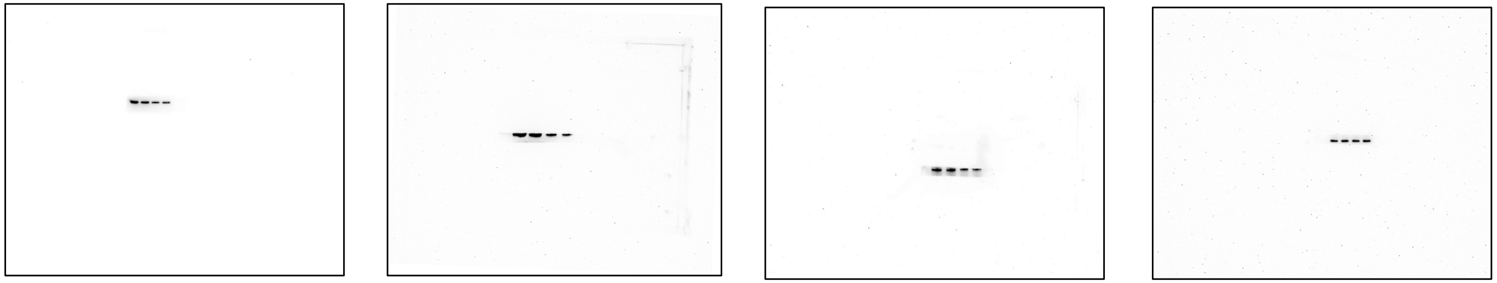

NR4A1 SOX9 S100A9 β-actin

Fig.3H

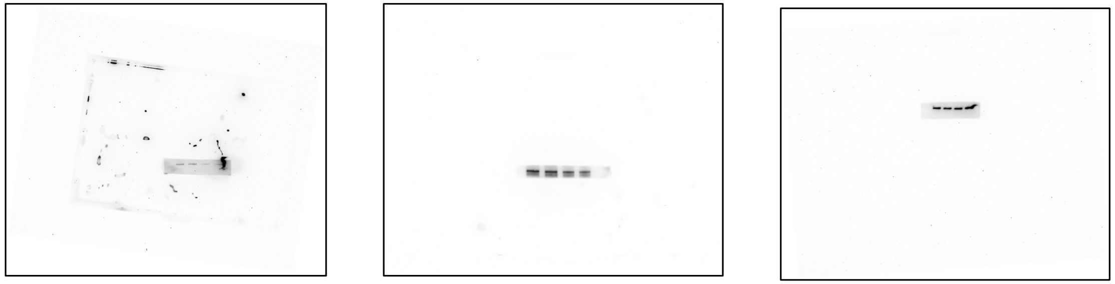

NR4A1 SOX9 β-actin

Fig.5C

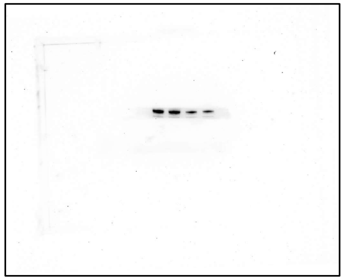

NR4A1

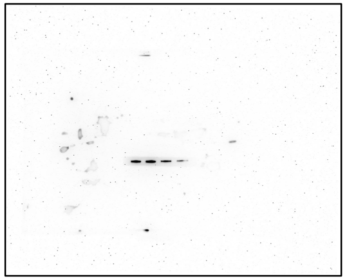

SOX9

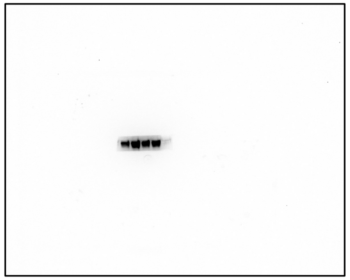

Lamin B2

Fig.5D: coIP-1

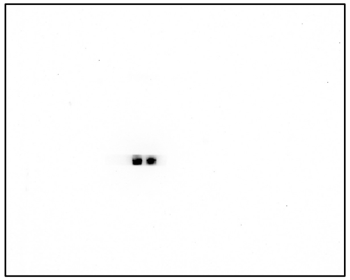

NR4A1

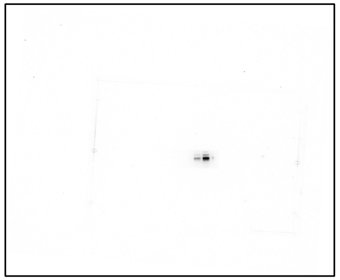

SOX9

Fig.5D: coIP-2

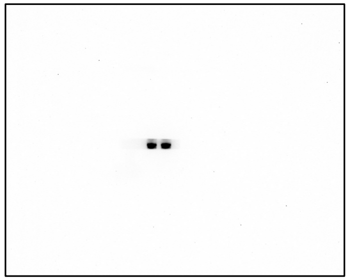

NR4A1

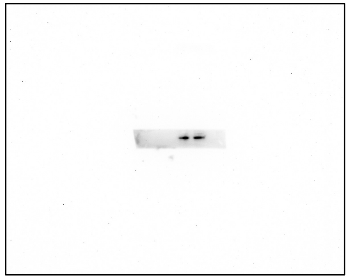

SOX9

Fig.6B

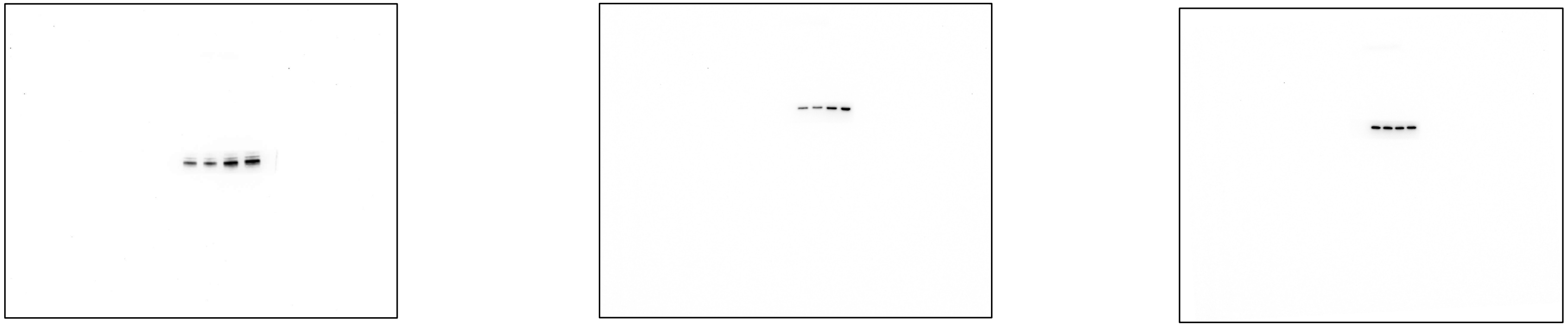

SOX9 S100A9 β-actin

Fig.6C

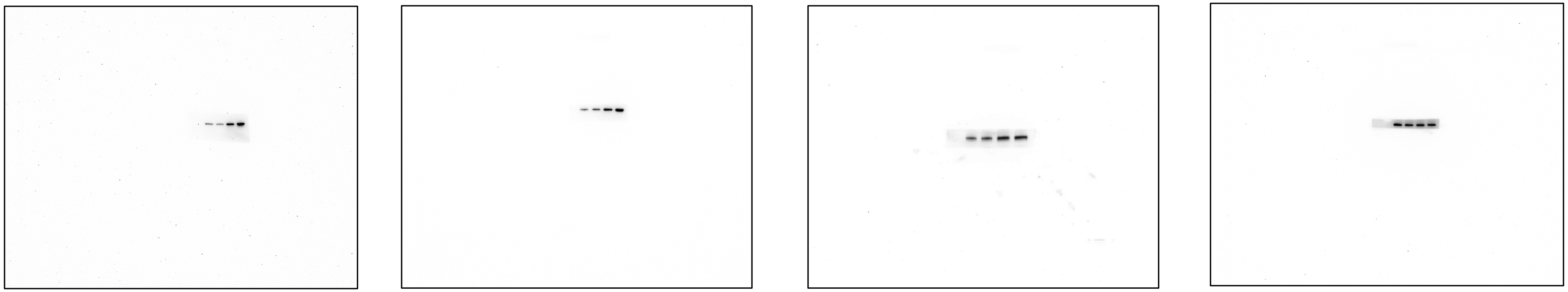

NEK7 NLRP3 C-Caspase 1 β-actin

Fig.7A

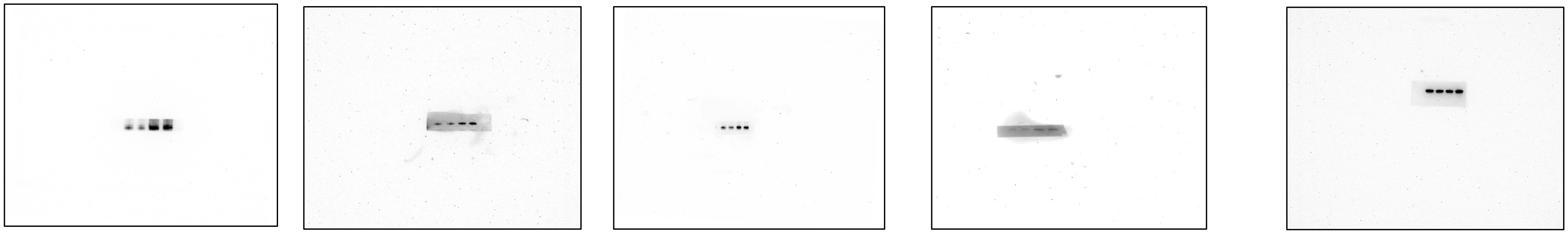

S100A9 NEK7 NLRP3 C-Caspase 1 β-actin

Fig.7D

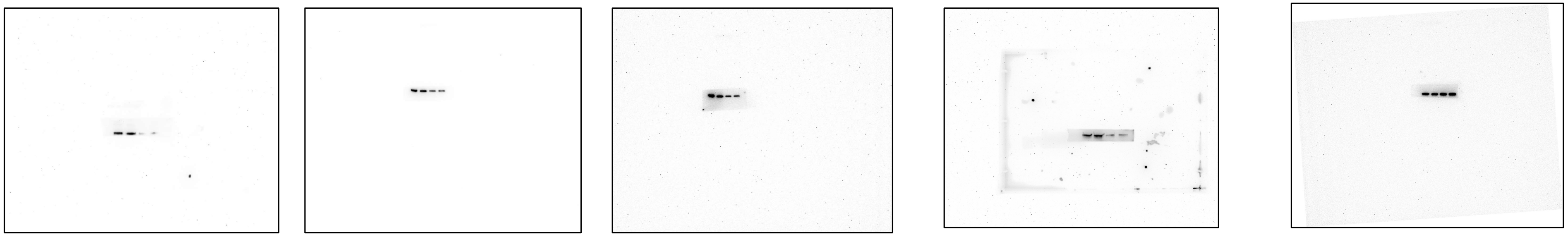

S100A9 NEK7 NLRP3 C-Caspase 1 β-actin
